# Signal analysis of whole-body shortening behavior in *Hirudo verbana*

**DOI:** 10.1186/1471-2202-13-S1-P14

**Published:** 2012-07-16

**Authors:** Benjamin J Migliori, Christopher Palmer, William Kristan

**Affiliations:** 1 Department of Physics, University of California San Diego, La Jolla, CA 92092, USA; 2 Department of Neuroscience, University of California San Diego, La Jolla, CA 92092, USA

## 

The European medicinal leech, *Hirudo verbana*, is a complex predator with a substantial repertoire of behaviors, most of which can be observed in the isolated nerve cord as “fictive” motor patterns. Each of the 21 midbody segmental ganglia of ~400 neurons is highly iterated and controls an individual body segment. Propagating behaviors (such as swimming, crawling, and shortening) rely on inter-ganglionic communications routed through connective nerves. However, for some observed behaviors, it is not known if the entire behavioral circuit is contained within each individual ganglion or if input from distal ganglia (> 2 segments away) plays a role in the resulting motor action and timing. Specifically in whole-body shortening (WBS), motor output is generated with left-right symmetry and propagates extremely rapidly towards the posterior of the animal, resulting in intense and rapid contraction of the body wall. Using *en passant* recordings (in which an extracellular electrode records all signals passing a specific location in the connective nerves) with extracellular motor neuron recordings, we have characterized the onset and latencies of WBS as a function of stimulation site. Our results indicate that WBS is primarily initiated locally and radiates outward with a fixed rapid propagation velocity. A possible explanation for the speed of WBS is the recruitment of the fast-conducting S cell network, which is known to conduct signals quickly enough to match the speed of WBS. However, our experiments reveal that lesioning Favre’s nerve to disrupt the S cell network results in a fixed delay past the lesion and no change to the propagation velocity. This suggests that pathways traveling through the lateral connectives may be re-recruiting the S cell network past the lesion. In order to identify the source of these neural signals, we use a novel application of the smoothed nonlinear energy operator to detect spikes and then class them by energy amplitude. The hierarchically classed spikes can then be removed from the data in an iterative process. This preprocessing improves our ability to separate sources in our highly overconstrained system. After separating our sources, superparamagnetic clustering is used to objectively sort spike sources. By measuring the variation in the timing of the sorted spike trains as a function of stimulus location and lesion presence, we hope to localize sources to specific ganglia. We will also use the observed patterns and timings to statistically characterize WBS for future experiments.

